# A Recombinant Subunit Based Zika Virus Vaccine Is Efficacious in Non-human Primates

**DOI:** 10.3389/fimmu.2018.02464

**Published:** 2018-11-08

**Authors:** Liana O. Medina, Albert To, Michael M. Lieberman, Teri Ann S. Wong, Madhuri Namekar, Eileen Nakano, Hanne Andersen, Jake Yalley-Ogunro, Jack Greenhouse, Stephen Higgs, Yan-Jang S. Huang, Dana L. Vanlandingham, Jaime S. Horton, David E. Clements, Axel T. Lehrer

**Affiliations:** ^1^Department of Tropical Medicine, Medical Microbiology & Pharmacology, John A. Burns School of Medicine, University of Hawai‘i at Mānoa, Honolulu, HI, United States; ^2^Bioqual Inc., Rockville, MD, United States; ^3^Department of Diagnostic Medicine/Pathobiology, Biosecurity Research Institute, College of Veterinary Medicine, Kansas State University, Manhattan, KS, United States; ^4^Hawaii Biotech, Inc., Honolulu, HI, United States

**Keywords:** zika virus (ZIKV), recombinant subunit, cynomolgus macaque, vaccine, CoVaccine HT™, microsphere immunoassay (MIA)

## Abstract

Zika Virus (ZIKV), a virus with no severe clinical symptoms or sequelae previously associated with human infection, became a public health threat following an epidemic in French Polynesia 2013–2014 that resulted in neurological complications associated with infection. Although no treatment currently exists, several vaccines using different platforms are in clinical development. These include nucleic acid vaccines based on the prM-E protein from the virus and purified formalin-inactivated ZIKV vaccines (ZPIV) which are in Phase 1/2 clinical trials. Using a recombinant subunit platform consisting of antigens produced in *Drosophila melanogaster* S2 cells, we have previously shown seroconversion and protection against viremia in an immunocompetent mouse model. Here we demonstrate the efficacy of our recombinant subunits in a non-human primate (NHP) viremia model. High neutralizing antibody titers were seen in all protected macaques and passive transfer demonstrated that plasma from these NHPs was sufficient to protect against viremia in mice subsequently infected with ZIKV. Taken together our data demonstrate the immunogenicity and protective efficacy of the recombinant subunit vaccine candidate in NHPs as well as highlight the importance of neutralizing antibodies in protection against ZIKV infection and their potential implication as a correlate of protection.

## Introduction

Zika virus (ZIKV) is an enveloped, single-stranded RNA virus within the Flavivirus genus, primarily transmitted by *Aedes* mosquito species. Although previously associated with asymptomatic or mild disease (1, 2), ZIKV is now emerging as causally associated with severe neurological and ophthalmological malformations in fetuses after congenital infection, as well as neurological disorders such as Guillain-Barré Syndrome in adults after recent ZIKV infections in French Polynesia (3, 4), and Brazil (5, 6). Due to the spread of the virus by the arthropod vector and secondary transmission through sexual contact (7), blood transfusions (8), and transplacental means (9, 10), the virus currently circulates in more than 80 countries and territories within Africa, Asia, and the Americas (11). In response, a strong global initiative to develop safe and effective countermeasures has engendered more than 30 preclinical vaccine candidates (12). Currently, there is no licensed vaccine to prevent disease associated with ZIKV infection; however, several ZIKV vaccine candidates have progressed to Phase 1/2 clinical trials, including DNA/mRNA, purified inactivated ZIKV, and measles virus-vectored vaccines.

The nucleic acid vaccine platform relies on the immunogenicity of the antigen generated through *de novo* synthesis in the recipient. The current ZIKV nucleic acid vaccines encode for the premembrane-envelope (prM-E) protein, with modifications flanking or within the transgene to improve antigen secretion, and have been shown to confer complete protection against ZIKV challenge in mice and non-human primates (NHPs) (13–17). Recently, three different Phase 1 clinical trials of a 3-dose DNA vaccine (NCT02840487, NCT02996461, and NCT02887482) have demonstrated favorable antibody responses in healthy recipients, but the vaccine candidates have yet to be proven effective in an international, and/or endemic setting (18, 19). Other Phase 1 trials of a purified, formalin-inactivated ZIKV vaccine (ZPIV) (NCT02963909, NCT02952833, and NCT02937233) have also shown protective potential in healthy recipients (20). While these vaccine candidates elicit seroconversion, it is unclear whether they are suitable for high-risk individuals (e.g., pregnant women, the elderly, and the immunocompromised).

Recombinant subunit immunogens that are properly folded and adjuvanted can be used to prime and boost the immune response against a specific pathogen when introduced into the recipient, and have one of the highest safety profiles. Multiple flavivirus vaccine candidates have been developed using an expression platform based on the *Drosophila* S2 insect cell expression system (21, 22). This expression platform has been shown to yield high quantities of secreted, conformationally authentic, monomeric E protein (23, 24). Experiments in mice and NHPs have demonstrated the ability of these recombinant proteins to generate high neutralizing antibody titers and to confer complete protection against homologous viral challenge (23, 25).

We have previously reported the production and purification of *Drosophila* S2 derived-ZIKV E protein, and evaluated its immunogenicity and efficacy in mice (26). This recombinant subunit candidate vaccine induced robust virus-specific antibody levels, including high neutralizing antibody titers. Furthermore, challenge studies in immunized, immunocompetent mice demonstrated inhibition of disseminated viral infection. In the present study, we evaluate the immunogenicity and protective efficacy of the ZIKV E protein vaccine candidate in cynomolgus macaques.

## Materials and methods

### Ethical statement

The investigators adhered fully to the “Guide for the Care and Use of Laboratory Animals” by the Committee on Care of Laboratory Animal Resources Commission on Life Sciences, National Research Council. Cynomolgus macaques (*Macaca fascicularis*) were housed at BIOQUAL Inc. (Rockville, MD). All macaque experiments were reviewed and approved by BIOQUAL's Animal Care and Use Committee. Mice were housed at The University of Hawai'i's John A. Burns School of Medicine (JABSOM) Laboratory Animal Facility (Honolulu, HI). Both facilities are accredited by the American Association for Accreditation of Laboratory Animal Care (AAALAC).

### Virus stock and cell culture

ZIKV, Puerto Rican Strain PRVABC59 stock, Dengue virus type 2 (DENV2) (Dakara strain) and West Nile Virus (WNV) (NY 99 Crow strain) were grown in Vero cells as previously described (26–28).

### Cynomolgus macaque vaccination, challenge, and blood collection

Expression of ZIKV E protein in the *Drosophila* S2 system and purification was done as previously published (26). Cynomolgus macaque studies were performed using two different vaccine formulations with different adjuvants: One formulation contained 25 μg of ZIKV E which was adjuvanted with 10 mg CoVaccine HT™ (BTG International Ltd, London, United Kingdom) and the second formulation used 50 μg ZIKV E protein with Alhydrogel® 85 at 1.2 mg of elemental aluminum (E.M. Sergeant Pulp and Chemical Co., Inc., Clifton, NJ). Both formulations were tested in female cynomolgus macaques (*n* = 4 for each formulation). The ZIKV E with CoVaccine HT™ formulation was tested in animals that were 8 years of age and the formulation containing ZIKV E with Alhydrogel® 85 was tested in animals that were between 4 and 12 years of age and weighed between 3 and 6 kg. All cynomolgus macaques were vaccinated intramuscularly (IM) in both legs (split dose) at days 0 and 21. Control animals that were part of the first formulation group received 25 μg of each of Sudan Virus (SUDV), Ebola Virus (EBOV), and Marburg Virus (MARV) glycoproteins (GP; control group, *n* = 4) at day 0 and PBS at day 21. Control animals challenged concurrently with the second formulation group received 25 μg of either SUDV GP or MARV GP adjuvanted with 10 mg CoVaccine HT™ at 0, 21, and 42 days. The control animals used in both studies were concurrently part of another, unrelated, study run to reduce animal numbers used due to ethical considerations. Pre-challenge serum and plasma samples were collected at days 0, 14, 21, 35, and 49. Challenge was performed on day 49 subcutaneously in the hind thigh with a non-lethal dose (10^4^ TCID_50_) of ZIKV Puerto Rican strain PRVABC59 in a volume of 1 mL PBS. Blood samples were collected daily for the following 7 days, then weekly until day 77. Plasma and/or serum samples were immediately processed and subsequently stored at −80°C prior to analysis.

### Viremia assays

Viremia was assessed either by quantitative RT PCR or plaque assay. For the RT PCR (conducted on NHP samples), RNA was isolated from 200 μL plasma using the QIAamp MinElute Virus spin kit (Qiagen, Frederick, MD). Extracted RNA was used for amplification using the SensiFAST Probe Lo-ROX One-Step Kit (Bioline BIO-78005, Taunton, MA) on a 7,500 Real-Time PCR system (Applied Biosystems, Foster City, CA). Primers and probe were designed to amplify a conserved region of the capsid gene from ZIKV BeH815744, as follows: Fwd: GGAAAAAAGAGGCTATGGAAATAATAAAG; Rev: CTCCTTCCTAGCATTGATTATTCTCA; Probe: AGTTCAAGAAAGATCTGGCTG. Primers and probe were used at a final concentration of 2 μM, and the following program was run: 48°C for 30 min, 95°C for 10 min, followed by 40 cycles of 95°C for 15 s and 1 min at 60°C. Assay sensitivity was 50 copies/mL.

Plaque assays (conducted on mouse serum samples collected post virus challenge) were performed by diluting serum and incubating with Vero cells at 37°C for 1 h. Cells were overlaid with M199 medium containing 1% agarose and incubated for 3 days at 37°C. A second overlay of 1% agarose in M199 containing 1.5% of a 0.33% solution of neutral red (Sigma-Aldrich, cat. no. N2889, St. Louis, MO) was added, and plaques were counted 24 h later. Limit of detection was 50 pfu/mL.

### Plaque reduction neutralization test (PRNT)

All PRNT assays were performed based on our standardized protocol (25) with slight modifications as detailed below. Prior to assay performance, sera from individual animals were heat-inactivated by incubation at 56°C for 30 min.

For the ZIKV PRNTs a series of 3 or 6 four-fold serial dilutions (for control or vaccinated animals, respectively), starting at 1:20 of each sample was prepared using M199 medium and incubated at 4°C overnight with media containing previously titrated virus in a 1:1 (v/v) ratio to generate approximately 50 plaques per well. The antibody-virus complex was then added to Vero cells in duplicate and incubated at 37°C for 1 h. Cells were overlaid with M199 containing 1% agarose and incubated for 24 h. A second overlay of 1% agarose in M199 containing 1.5% of a 0.33% solution of neutral red was added, and plaques were counted 24 h later.

For the DENV and WNV PRNTs a series of 3 or 6 two or four-fold dilutions, starting at 1:5 (for WNV) or 1:10 (for DENV) of each sample was prepared using M199 medium, and incubated for 30 min at room temperature with media containing previously titrated virus in a 1:1 (v/v) ratio to generate approximately 50 plaques per well. The antibody-virus complex was then added to Vero cells in duplicate and incubated at 37°C for 1 h. Cells were overlaid with M199 containing 1% agarose and incubated for either 48 h (WNV) or 72 h (DENV). A second overlay of 1% agarose in Dulbecco's phosphate buffered saline (DPBS) containing 1.5% (WNV) or 2% (DENV) of the 0.33% neutral red solution was added, and plaques were counted 24–48 h later.

PRNT_50_ values, the serum dilutions yielding 50% virus neutralization, were generated using a variable-slope sigmoidal dose response computer model (Prism, Graphpad Software, San Diego, CA). Serum dilutions were calculated after addition of an equal volume of virus suspension. Thus, serum diluted 1:5 and then mixed with virus in a 1:1 ratio yields a 1:10 dilution. PRNT data shown for all viruses is derived from serum collected at day 35 (14 days after the booster immunization).

### Coupling of microspheres with recombinant antigens and microsphere immunoassay (MIA)

The coupling of microspheres with ZIKV E protein, the conduct of the MIA, and the derivation of EC_50_ antibody titers were performed as described previously (26).

### Passive transfer studies in BALB/c mice

BALB/c mice were bred in colonies at JABSOM from original stocks obtained from Taconic Biosciences, Inc. (Hudson, NY). One hundred microliters of plasma taken from cynomolgus macaques 2 weeks after the second immunization with CoVaccine HT™ adjuvanted ZIKV E (day 35) were injected intraperitoneally (IP) into 3 groups of 12 sex balanced, 6–7 months old mice one day prior to challenge with 100 pfu of live ZIKV via the tail vein. Serum collection by tail vein bleeds was performed 6 h after the serum transfer, and blood collection by cardiac puncture was performed at day 3 after challenge for six of the animals in each group and 2 weeks after challenge for the remaining animals. ZIKV E-specific IgG antibody titers respective to both animal species, assayed by MIA, and ZIKV neutralizing titers were determined on serum samples.

### Statistical analysis

Determination of differences between ZIKV E specific IgG titers was done using an unpaired *t*-test with Welch's correction. Determination of statistical significance in the number of viremic days between vaccinated animals and control group animals in the NHP challenge experiments was done using the Fisher exact probability test. Differences in viremia and IgG titers between groups of mice in the passive transfer experiment was assessed for statistical significance using the ANOVA for multiple comparisons (Prism, Graphpad Software, San Diego, CA). *P* < 0.05 was considered significant.

## Results

### Immunogenicity in cynomolgus macaques

**Figure 1:**
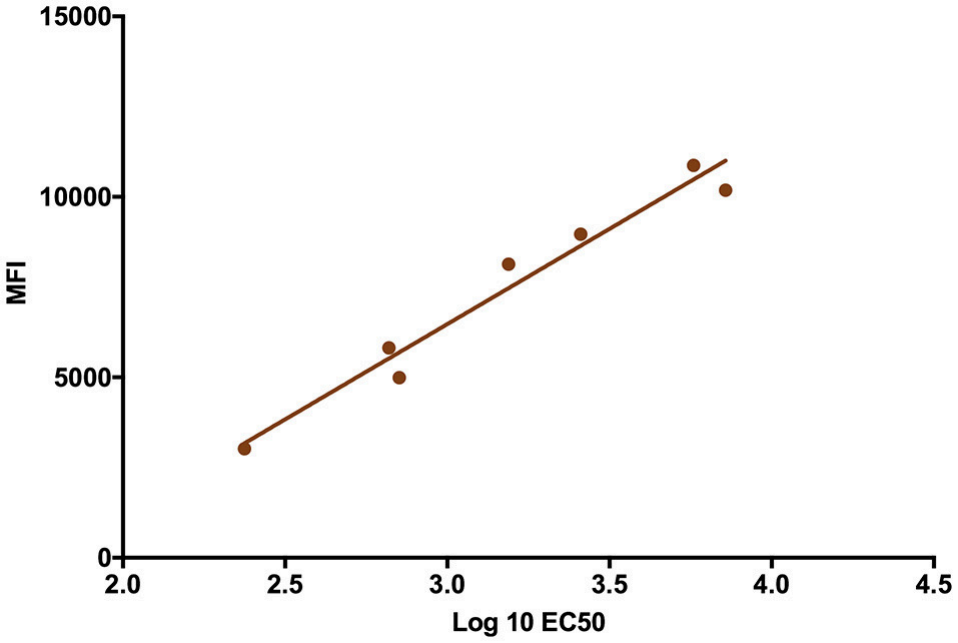
Median Fluorescence Intensity (MFI) Correlation with EC_50_. The log EC_50_ values of seven NHP serum samples were determined by MIA and plotted against the MFI values obtained from these sera at a dilution of 1:1,000 for each serum sample (*R*^2^ = 0.960).

**Figure 2:**
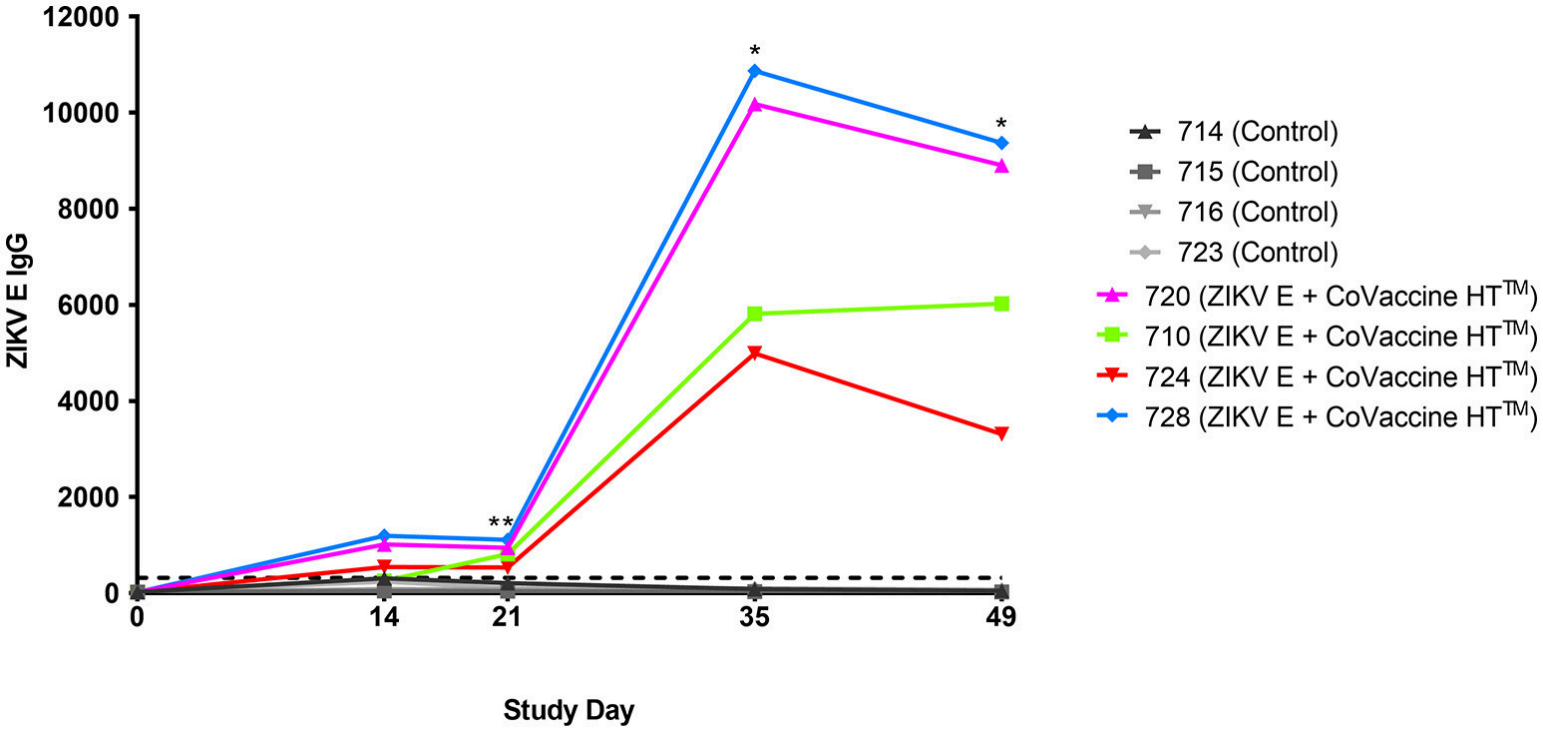
Immunogenicity of recombinant ZIKV E protein in cynomolgus macaques. Animals were given 25 μg ZIKV E protein with CoVaccine HT™ as an adjuvant at day 0 and 21. The control group received unrelated glycoproteins at day 0 and PBS at day 21. Blood was collected at various points throughout the vaccination schedule. Individual animal's ZIKV E specific IgG MFI of serum from the vaccinated and control groups are depicted. The negative assay cutoff is shown by the dotted line. It was calculated by taking the mean value of the pre-vaccination samples (day 0) of all 8 animals and adding 3 standard deviations. Vaccinated animals had significantly higher ZIKV E specific IgG titers as compared to controls at days 21, 35, and 49 (^*^p < 0.05, ^**^p < 0.01).

The validity of single point MIA values as accurate determinants of antibody titers was established by correlation of the EC_50_ values obtained on multiple serum samples with varying ZIKV E specific antibody titers with their MFI values at a single dilution (1:1,000). Linear regression between the MFI at the 1:1,000 serum dilution and the EC_50_ of NHP samples with different IgG titers shows a good correlation (*R*^2^ = 0.960; Figure 1), allowing single point MFI values to be used as an accurate measurement of IgG titers. Therefore, IgG antibody titers are shown as MFI at the 1:1,000 dilution for all subsequent assays. The immunogenicity of the tested candidate vaccine formulation in NHPs was assessed by measuring ZIKV E protein-specific IgG levels in the serum of vaccinated and control NHPs using MIA (Figure 2). Animals in the vaccine group (CoVaccine HT™ adjuvanted) showed a ZIKV E specific IgG response by day 14 post immunization with titers rising after the second dose at day 21 and remaining high until the challenge at day 49. Similar IgG titers by MIA were seen in the animals receiving the ZIKV E with Alhydrogel® 85 formulation (Supplementary Figure 1). High ZIKV neutralizing antibody titers were found at day 35 in three of the four vaccinated animals receiving ZIKV E with CoVaccine HT™ (Table 1). One animal (724) had a low neutralizing antibody titer despite showing a high level of antigen binding antibodies (determined by MIA). All four animals receiving the ZIKV E with Alhydrogel® 85 developed high neutralizing antibody titers. None of the control animals developed ZIKV neutralizing antibody titers. Neutralizing antibody assays showed variability between vaccinated animals in cross-neutralization with other flaviviruses. Of the four vaccinated animals in the first formulation group (ZIKV with CoVaccine HT™), animal 720 had detectable neutralizing antibodies to DENV2 and WNV, with PRNT_50_ values of 1,186 and 392, respectively, and animal 728 had PRNT_50_ titers against DENV2 and WNV of 118 and 30, respectively (Table 1).

**Table 1:** Serum PRNT_50_ titers at day 35 of all animals against ZIKV, DENV2, and WNV.

**Experiment 1^a^**	**Animal ID**	**ZIKV PRNT_50_**	**DENV2 PRNT_50_**	**WNV PRNT_50_**	**Experiment 2^b^**	**Animal ID**	**ZIKV PRNT_50_**	**DENV2 PRNT_50_**	**WNV PRNT_50_**
Vaccinees	710	5,242	134	< 20	Vaccinees	6,749	4,558	65	24
	720	40,446	1,186	392		C29573	4,632	106	< 20
	724	48	< 20	< 20		C30730	2,420	71	59
	728	4,866	118	30		6,751	2,406	201	24
Controls	714	< 40	< 20	< 20	Controls	6,750	< 40	< 20	< 20
	715	< 40	< 20	< 20		6,752	< 40	< 20	< 20
	716	< 40	< 20	< 20		6,739	< 40	< 20	< 20
	723	< 40	< 20	< 20		6,744	< 40	< 20	< 20

a *Vaccine recipients in experiment 1 received ZIKV E with CoVaccine HT™*.

b *Vaccine recipients in experiment 2 received ZIKV E with Alhydrogel® 85*.

Recipients of the Alhydrogel® 85-adjuvanted formulation developed more consistent, but lower, cross-neutralizing antibody responses. All four vaccinated animals had a DENV cross-neutralizing response with PRNT_50_ titers in the range of 65–201 and three of the four vaccinated animals showed WNV cross-neutralizing antibodies with PRNT_50_ titers in the range of 24–59.

### Protection against viremia

**Figure 3:**
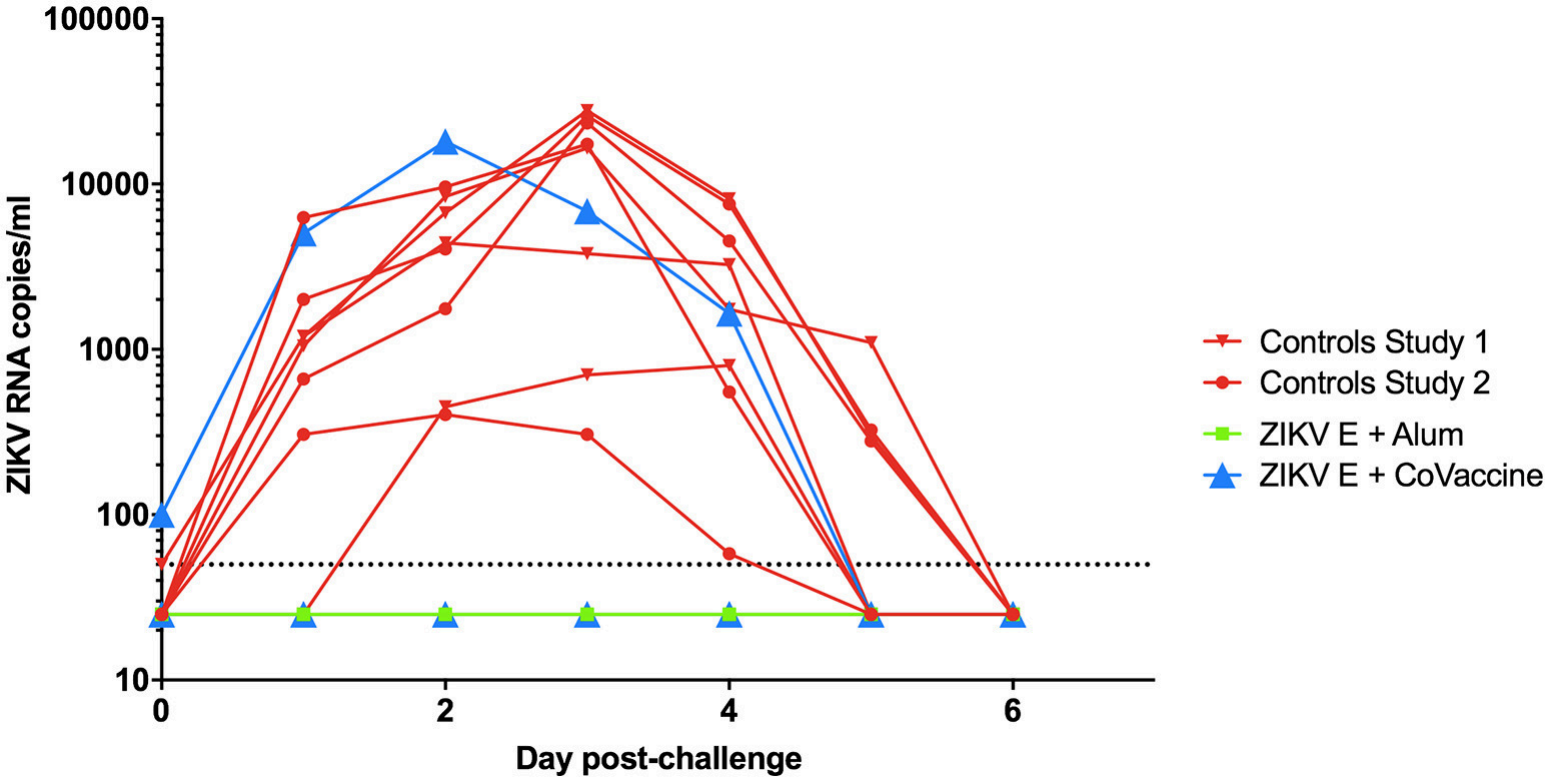
Viremia in vaccinated and control cynomolgus macaques challenged with ZIKV. The data from both experiments are combined in this figure. Animals were challenged at day 49 of both experiments. Blood was taken daily for 7 days post challenge, and viremia was measured using quantitative RT-PCR. Limit of detection was 50 copies/ml of plasma, shown by the dotted line. Vaccinated animals had a significantly reduced number of viremic days compared to control animals (*p* < 0.0002; Alum, Alhydrogel® 85).

After challenge with ZIKV on day 49, virus RNA was detectable in both groups of control animals starting 1 day after challenge with peak viremia being seen between days 2 and 4 (Figure 3). In the ZIKV E with CoVaccine HT™ vaccine group, three of the four animals had undetectable viremia on all days, while animal 724, which developed significantly lower neutralizing antibody titers showed viral RNA levels similar to the control animals. In the group that received ZIKV E with Alhydrogel® 85, all four animals were completely protected against viremia following viral challenge. These results demonstrate that infection of the animals was successful and that both ZIKV vaccine candidate formulations had the ability to elicit significant levels of protection, 75% for ZIKV E with CoVaccine HT™, and 100% for ZIKV E with Alhydrogel® 85.

### Plasma from ZIKV E immunized cynomolgus macaques protects mice from viral challenge

**Figure 4:**
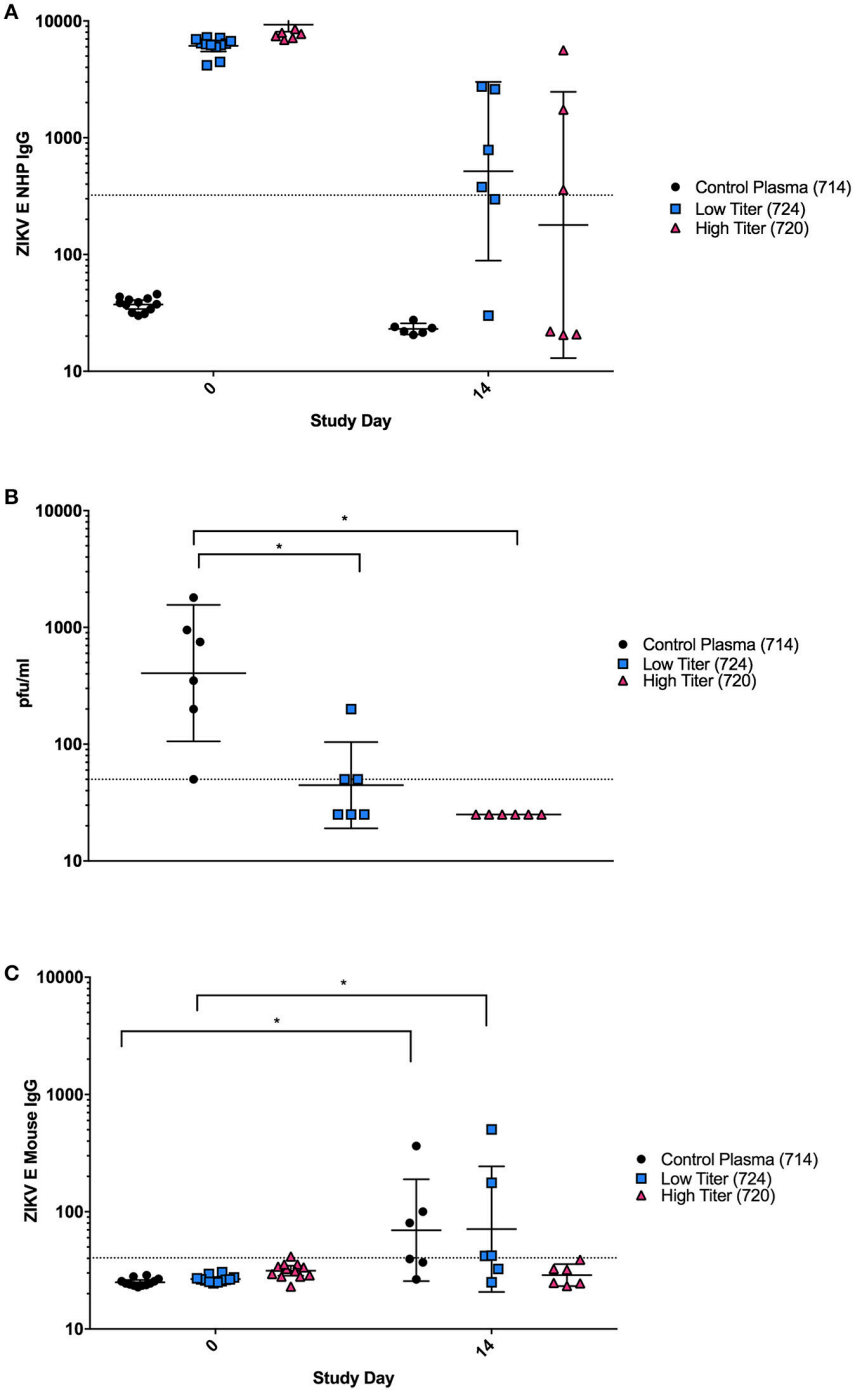
Passive Protection in BALB/C mice using NHP plasma. **(A)** Cynomolgus ZIKV E specific IgG titers expressed as MFI from serum at day 0 (6 h after passive transfer of plasma) and day 14 after transfer. The negative assay cutoff was calculated by taking the mean value of negative control samples and adding 3 standard deviations and is shown by the dotted line. Differences between mice receiving control plasma and mice receiving low or high titer plasma were significant (*p* < 0.0001) at day 0, but not at day 14. **(B)** Viremia from six individual mice from each group at day 3 post-infection was determined using a standard plaque assay on Vero cells. Limit of detection is 50 pfu/mL, shown by the dotted line. Data points below the level of detection are depicted as 25. Differences between mice receiving control plasma and mice receiving low titer or high titer plasma were significant (^*^p < 0.05). **(C)** Mouse ZIKV E specific IgG titers expressed as MFI. The negative cutoff was calculated by taking the mean value of negative control samples and adding 3 standard deviations and is shown by the dotted line. Differences between days 0 and 14 were significant (*p* < 0.05) in animals receiving control and low titer serum, but not high titer serum. **(A–C)**: Error bars represent the Geometric Mean Titer (GMT)+/−95% Confidence Intervals. Statistical differences were assessed using the ANOVA with multiple comparisons.

Although the PRNTs show that the antibodies elicited in the vaccinated NHPs were capable of neutralizing virus *in vitro*, we wanted to determine if these antibodies were also sufficient to neutralize virus *in vivo* and protect against ZIKV challenge. This was of particular interest due to the differences observed in binding vs. neutralizing antibody responses observed in animal 724. Plasma from three individual NHPs from the ZIKV E with CoVaccine HT™ group was injected IP into groups of 12 mice: (1) an unvaccinated control animal with no ZIKV neutralizing activity (714), (2) a vaccinated animal with low neutralizing titers (724) and (3) one with high neutralizing titers (720). Blood was collected from mice 6 h post-transfer to determine the levels of NHP and mouse anti-ZIKV E IgG by MIA (Figure 4). Mice were challenged the following day. On day 0, after passive transfer but before viral challenge, titers of ZIKV E specific NHP IgG were similar for the low and high titer mouse groups, and undetectable in all mice receiving the naïve plasma (Figure 4A). By day 14, levels of NHP IgG had dropped for low and high titer recipients and remained undetectable in mice that received naïve plasma. Despite the similar levels of binding antibodies present in the low and high titer mouse groups, three of the six mice that received low titer NHP serum developed viremia after challenge, demonstrating only partial protection, while the mice that received the high titer plasma were completely protected (Figure 4B). These results were supported by the mouse specific IgG titers from mouse sera (Figure 4C). At day 0, none of the mice showed any murine IgG titers against ZIKV E, however, by day 14 post challenge, mice in the naïve and some animals in the low titer group had increased levels of ZIKV E-reactive murine IgG indicating successful virus replication in these groups. As IgG titers against the E protein were only seen in mice of the naïve and low titer groups, this strongly suggests that viremia in the high titer group did not develop or was extremely short-lived, not lasting long enough for IgG antibodies to develop. This result therefore suggests that the high titer NHP serum had sufficient neutralizing antibodies to confer protection from viremia while the low titer serum did not contain enough virus neutralizing capacity despite showing similar antigen-binding IgG titers.

## Discussion

Many strategies have been used in the development of efficacious candidate flavivirus vaccines, including recombinant subunit platforms (23, 25, 29–32). Vaccines against DENV and WNV using recombinant subunits produced in *Drosophila* S2 cells have successfully undergone Phase 1 clinical trials (NCT00936429, NCT01477580, and NCT00707642). The tetravalent recombinant subunit DENV vaccine has proven to be safe and immunogenic, and protects NHPs from viremia (23, 31, 33). This report provides the first documentation of the development of a recombinant subunit vaccine that affords effective protection against ZIKV infection in NHPs after only two doses of vaccine. It has been well established that humoral immunity plays a major role in protection against flavivirus infection, with the E protein as the major antigenic target (30, 34–36). Recombinant protein subunit based vaccines are safe and highly immunogenic when paired with the correct adjuvant. For these studies we selected Alhydrogel® 85, a well characterized and effective adjuvant and CoVaccine HT™ which we have previously demonstrated to be efficacious when used for protein subunit vaccines (26, 37). Higher levels of antigen were used in the Alhydrogel® 85 formulation based on previous experience with this adjuvant. Using the *Drosophila melanogaster* Schneider S2 cell-based expression system to produce ZIKV E protein, we have demonstrated protection using either CoVaccine HT™ or Alhydrogel® 85 as adjuvant. These vaccine formulations were previously tested by our group in a mouse model, using immunocompetent Swiss Webster, BALB/c, and C57BL/6 mouse strains (26). Our studies showed that both vaccine formulations were able to elicit robust antigen binding and neutralizing antibody titers after two doses, and protect against viral replication after challenge with ZIKV. We have chosen to further test the ZIKV E formulations in cynomolgus macaques, in which it has been previously demonstrated that ZIKV PRVABC59 replicates robustly and with very similar kinetics and duration to what is seen in rhesus macaques, albeit with slightly lower peak viral titers (38). A cynomolgus macaque ZIKV model is also an appropriate choice due to its ability to provide a close comparison to human viral pathogenesis and immune response both to natural infection and vaccination (39). In this model the recombinant subunit vaccine candidates were found to elicit binding IgG antibody titers against ZIKV E protein by day 14 after the first vaccine dose which rose after a second vaccination on day 21, a response that lasted until ZIKV challenge at day 49 (Figure 2) demonstrating the immunogenicity of the vaccine candidate. ZIKV neutralizing antibody titers were also found to be high for three of the four vaccinated animals in the ZIKV E with CoVaccine HT™ group and all vaccinated animals in the ZIKV E with Alhydrogel® 85 group (Table 1). Animal 724 showed lower ZIKV neutralizing antibody titers compared to other animals, despite the presence of comparable antigen binding IgG titers. Viral RNA after ZIKV challenge in all animals with high neutralizing (PRNT_50_) titers was undetectable (Figure 3) throughout the challenge period demonstrating a correlation between the neutralizing antibodies elicited by vaccination and protection against peripheral viral infection. The animal that had a low PRNT_50_ titer (animal 724) developed viremia similar to the controls, and the viral kinetics agreed with what has been seen in cynomolgus macaques in previous experiments (38). These data highlight the importance of high neutralizing antibody titers which have been suggested as a correlate for efficacy in flavivirus vaccine development (40, 41) and which have been shown to prevent ZIKV transmission to fetuses in a pregnant mouse model (41). The high levels of ZIKV E binding IgG but low PRNT_50_ titer in animal 724, which showed breakthrough viremia, correspond to the development of antibodies that are capable of binding ZIKV E but are incapable of virus neutralization; therefore, these data support the use of neutralizing antibodies as a correlate of protection. Other ZIKV vaccine studies done in rhesus macaques have shown similar results with viremia being seen in animals with comparatively low neutralizing titers after receiving a DNA vaccine expressing ZIKV prM and E protein or an mRNA vaccine encoding ZIKV prM and E protein, while other animals with higher neutralizing titers were protected (14, 29). A comprehensive overview of these vaccine platforms and their performance in different animal models is covered in the recent review by Poland, et al. (42).

Interestingly, our recombinant subunit ZIKV E vaccine elicits antibodies that in some animals are capable of cross-neutralizing other flaviviruses (Table 1). While WNV but not DENV cross-binding antibodies were present in all vaccinated animals (data not shown), PRNT data showed that 7 of the 8 vaccinated animals had antibodies cross-neutralizing DENV2 and 5 of the 8 animals had antibodies cross-neutralizing WNV (Table 1). Of course, the question whether animal 724 might have had antibodies from a previous flavivirus infection resulting in an *in vivo* manifestation of antibody-dependent enhancement of infection (ADE) presented itself. However, this animal at the beginning of the study had no neutralizing antibodies for any of the tested flaviviruses (data not shown), and despite a high titer of ZIKV E binding antibodies present before challenge, it had the lowest virus neutralizing titers in the vaccinated group. ADE is a concern in the field of ZIKV vaccine development and experiments done in immunocompromised and wild type mice have yielded inconsistent results (35, 43, 44). Studies using rhesus macaques have shown that preexisting immunity to DENV does not enhance the pathogenesis of ZIKV infection and may in fact shorten the duration of viremia (45, 46); however, preexisting immunity to ZIKV may result in enhanced infectivity in a subsequent DENV infection (47). Furthermore, analysis of patients infected with ZIKV has also shown that previous infection with DENV had no effect on the severity of the ZIKV infection (46). Our experiments showed no evidence of virus enhancement due to sub-neutralizing concentrations of antibody and we attribute the lack of a neutralizing response in animal 724 to biological variation among these outbred animals. While an in-depth analysis of the potential antibody-dependent enhancement as frequently discussed in the context of flavivirus infections is outside the scope of this report, the topic remains of interest for future studies.

A passive transfer experiment shed further light into the importance of neutralizing antibodies. Mice receiving plasma from the control NHP had no detectable cynomolgus IgG binding ZIKV E antibodies while the mice that received low and high neutralizing titer NHP plasma had high pre-challenge IgG titers demonstrating a successful transfer. Mice that received plasma from the unvaccinated control macaque uniformly showed viremia after challenge, while three of the six mice receiving the low titer plasma and none of the mice that received the high titer plasma developed viremia. Mouse IgG titers rose only in sera of mice that received control and low titer plasma. The development of IgG titers in the low titer and control recipient mice is consistent with the viremia seen in these groups, while the lack thereof in the group that received high titer plasma can be explained by a lack of viral replication in these animals. These data correlate well with other studies that have demonstrated that protection against several different flaviviruses in mouse models can be achieved through antibodies alone (35, 36, 48, 49, 50, 51, 52). In the case of our passively protected mice, lack of viremia in the high titer antibody recipients and a failure to develop ZIKV E-specific IgG suggest that full protection against viral replication was achieved, and demonstrates the ability of the recombinant ZIKV E vaccine to induce a completely protective humoral immune response. Although many ZIKV infection studies employ the use of immunocompromised mouse models, particularly when exploring pathogenesis, we feel that these models would not be appropriate for our studies as they are not a good representation of ZIKV infection in the general human population (41, 53) and would only be applicable to a subset of immunocompromised individuals. The use of older mice in the experiments described here, however, may partially address the broader population as immunosenescence generally increases the susceptibility to infection. Taken together these results suggest that recombinant ZIKV E subunits could be a safe and efficacious option for the prevention of ZIKV infection in humans. Importantly, as IgG crosses the placental barrier, virus neutralizing antibodies produced in response to a safe vaccine administered to a pregnant mother could also protect the unborn child from the detrimental neurovirulence of ZIKV infection.

## Author contributions

LM, AT, DC, ML, and AL: conceptualization; HA, JY-O, JG, SH, Y-JH, and DV: NHP studies, sample preparation, viremia analysis; LM, AT, and TW: mouse study; LM, AT, TW, MN and EN: sample acquisition and analysis; LM, AT, ML, TW, and AL: experimental analysis; AL: project administration; LM and ML: visualization and analysis; LM, AT, ML, TW and AL: writing—original draft; All authors: writing—review and editing.

### Conflict of interest statement

JH and DC are employees of Hawaii Biotech, Inc. and hold stock and/or stock options in the company. The remaining authors declare that the research was conducted in the absence of any commercial or financial relationships that could be construed as a potential conflict of interest.
