# Supplementary material for: A Recombinant Subunit Based Zika Virus Vaccine Is Efficacious in Non-human Primates
Source: Front Immunol. 2018 Nov 8;9:2464. doi: 10.3389/fimmu.2018.02464 (PMC6236113; doi:10.3389/fimmu.2018.02464)

**Supplementary Figure 1.** Immunogenicity of recombinant ZIKV E protein in cynomolgus macaques given 50 μg ZIKV E protein adjuvanted with Alhydrogel® 85 on days 0 and 21. The control group received unrelated glycoproteins at day 0, 21, and 42 adjuvanted with CoVaccine HT™. Individual animal’s ZIKV E specific IgG MFI of serum from the vaccinated and control groups are depicted. The negative assay cutoff is shown by the dotted line. It was calculated by taking the mean value of the pre-vaccination samples (day 0) of all 8 animals and adding 3 standard deviations. Two control animals, 6750 and 6739, showed increased reactivity following a third vaccination on day 42 with the unrelated glycoproteins in adjuvant.


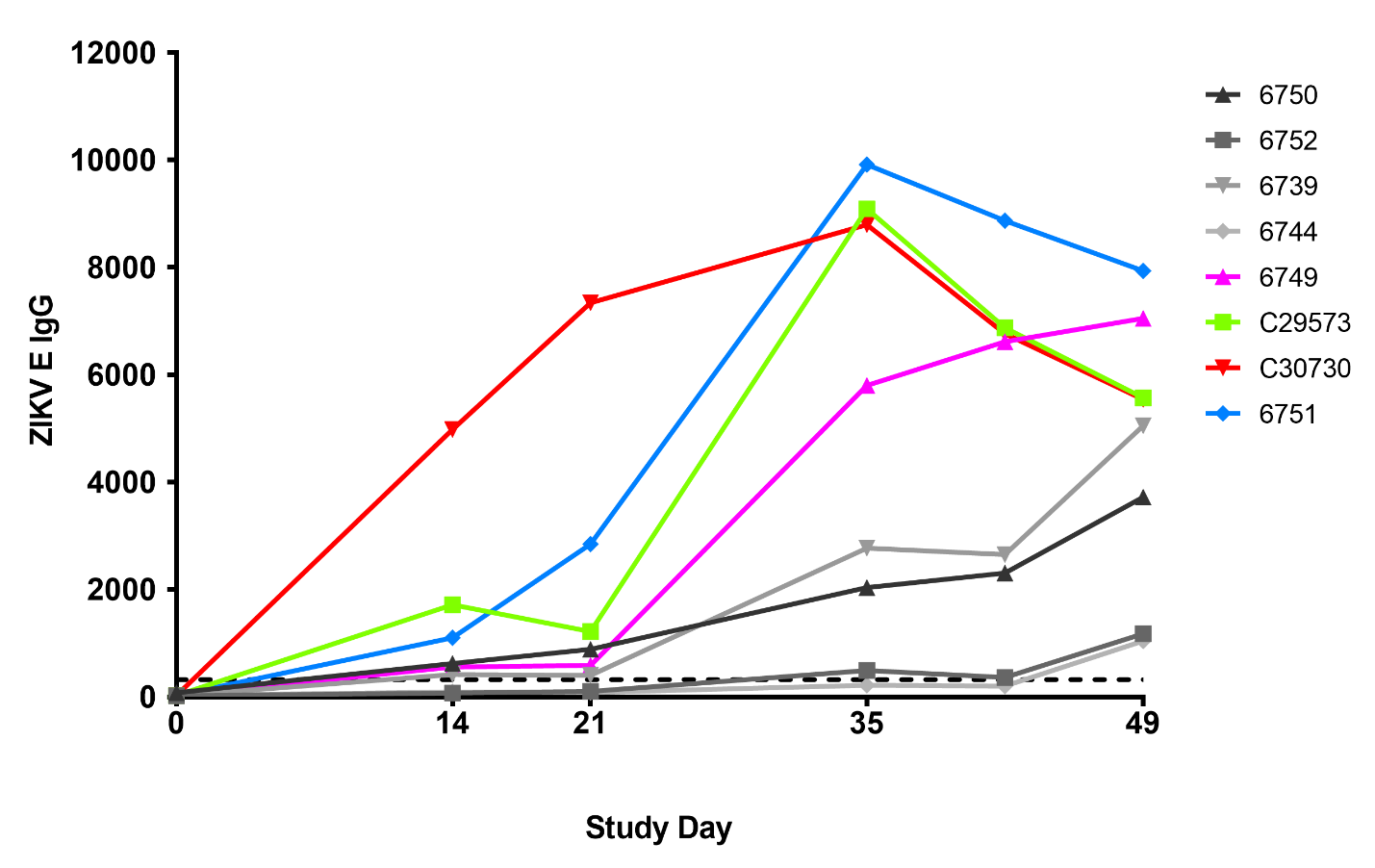

Supplement: Supplementary file 1 [file Data_Sheet_1.docx]
